# Efficacy and Safety of Tranexamic Acid in Reducing Blood Loss of Lower Extremity Osteotomy in Peri‐acetabulum and High Tibia: A Systematic Review and Meta‐analysis

**DOI:** 10.1111/os.12515

**Published:** 2019-08-28

**Authors:** Ru‐zhan Yao, Wei‐qiang Gao, Bing‐wu Wang, Guang‐lin Wang, Cheng‐xi Wu, Yi‐da A‐mu

**Affiliations:** ^1^ Department of Orthopaedics West China Hospital, Sichuan University Chengdu China; ^2^ Department of Spinal Surgery Weifang People's Hospital Weifang China; ^3^ Deparment of Orthopedics The People's Hospital of Guang'an City Sichuan China; ^4^ Deparment of Orthopedics Chengdu Integrated Traditional Chinese and Western Medicine Hospital, The First People's Hospital of Chengdu Sichuan Province Sichuan Sheng China

**Keywords:** Blood loss, High tibial osteotomy, Meta‐analysis, Periacetabular osteotomy, Tranexamic acid

## Abstract

**Objective:**

To assess the efficacy of tranexamic acid (TXA) in reducing total blood loss and transfusion, and the risk of thromboembolic events in patients undergoing periacetabular osteotomy (PAO) and high tibial osteotomy (HTO).

**Methods:**

A systematic literature search was performed using PubMed, the Cochrane Central Register of Controlled Trials (CENTRAL), Embase (Ovid), Medline (Ovid), and Web of Science. ClinicalTrials.gov, American Academy of Orthopaedic Surgeons (AAOS), and Orthopaedic Trauma Association (OTA) conference proceedings were also searched to gain more eligible studies. The primary outcome measure was total blood loss and the blood transfusion rate of the TXA group versus control. The meta‐analysis was conducted using the RevMan 5.3 and Stata 14.0 software.

**Results:**

A total of six studies were included involving 665 patients. Three studies were PAO, and the other three were HTO. The total blood loss in PAO (WMD, −330.49; 95% *CI*, −390.16 to −270.83; *P* < 0.001) and HTO (WMD, −252.50; 95% *CI*, −356.81 to −148.18; *P* < 0.001) and hemoglobin decline (WMD, −0.74; 95% *CI*, −1.09 to −0.38; *P* < 0.001) were significantly less in the TXA group than in the control group. TXA could reduce transfusion rates in PAO (RR, 0.26; 95% *CI*, 0.09 to 0.75; *P* = 0.01) but had no effect on HTO (RR, 0.20; 95% CI, 0.01 to 4.10; *P* = 0.30). The wound complications (RR, 0.62; 95% *CI*, 0.13 to 2.94; *P* = 0.54) had no significant difference between TXA and control groups.

**Conclusions:**

This meta‐analysis demonstrated that TXA reduces total blood loss and hemoglobin decline in patients undergoing PAO and is safe, but it has little benefit in regard to reducing transfusion rates or wound complications in HTO, so TXA might be unwarranted for routine use for HTO.

## Introduction

Periacetabular osteotomy (PAO) is a very effective surgery to treat pre‐arthritic symptomatic acetabular dysplasia, and its clinical and radiographic outcomes are excellent^1^. Blood loss in patients undergoing PAO is a major source of morbidity, even causing death, especially for PAO. However, this surgery needs to dissect the ilium and ischium, which are rich vascular territories, and the duration of surgery is much longer than an ordinary operation. Heavy blood loss is caused by these factors^2^. Lee *et al*. reported that the blood loss after PAO was up to 3900 mL^3^. Up to 45% of patients undergoing PAO require blood transfusion (either autologous or allogeneic transfusion)^4^. Blood transfusions may increase the risk of bacterial infection and have been found to cost over $1731 per admission^5^. Most important of all, there is currently no guideline for the management of blood loss or the use of pharmacological agents during PAO^6^.

High tibial osteotomy is a well‐established option for medial compartment osteoarthritis or spontaneous osteonecrosis of the knee^7^. However, the operation can induce extensive bone bleeding as a result of the bone gap and the releasing of extensive soft tissue^8^. Lack of soft tissue coverage around the proximal medial tibial surface creates some disadvantages, such as wound hematoma, delayed union, and superficial skin infections^9^, ^10^. It is reported that approximately 6% patients suffer from wound complications after HTO^11^, ^12^. Therefore, reducing bleeding intraoperation and postoperation could reduce wound complications and improve the curative effect of the operation.

Tranexamic acid is a synthetic lysine analogue that competitively inhibits the activation of plasminogen to plasmin, thus temporarily avoiding the dissolution and degradation of fibrin clots by plasmin^13^. Furthermore, TXA blocks the dispersion of platelets, which can promote the clot formation^14^. TXA has demonstrated good efficacy in reducing blood loss and improving safety in trauma surgery, arthroplasty, maxillofacial surgery, and cardiac surgery^5^, ^15^, ^16^, ^17^, ^18^, ^19^, ^20^.

Despite the evidence of the benefits of TXA, it is not routinely used in PAO or HTO. There is little evidence to prove the efficacy or safety of TXA in PAO or HTO. Many surgeons hesitate to administer TXA in PAO or HTO because the effects and safety are not certain. Therefore, we conducted the first meta‐analysis to systematically review the efficacy in reducing total blood loss and transfusion rate and the association between TXA and thromboembolic events in patients undergoing PAO and HTO.

## Methods

This meta‐analysis was performed according to the Preferred Reporting Items for Systematic Reviews and Meta‐Analyses (PRISMA) guidelines. Ethics approval and consent to participate were not needed, as this meta‐analysis is based on previous published studies. The review protocol was registered online, and the unique identifying number is Review Registry 593.

### *Literature Search*

The system retrieval for clinical trials was performed across various databases using Mesh terms supplemented with free words. The databases included PubMed (1968 to October 2018), the Cochrane Central Register of Controlled Trials (October 2018), Embase (1974 to October 2018), Medline (1968 to October 2018), and Web of Science (1990 to October 2018). We also searched for unpublished data using the American Academy of Orthopaedic Surgeons (AAOS, October 2018) and the Orthopaedic Trauma Association (OTA, October 2018) conference proceedings. Finally, ClinicalTrials.gov (October 2018) was searched. Two researchers independently performed the comprehensive study search using the search terms: (tranexamic acid OR TXA OR AMCHA) AND (periacetabular osteotomy OR tibial osteotomy OR PAO OR HTO) AND blood loss. There was no language or regional limitation. A medical librarian designed and supervised the search process, and before the literature search a statistical analysis plan was made.

Search strategy of PubMed and Embase:

#1 periacetabular osteotomy OR PAO OR periacetabular surgery

#2 high tibial osteotomy OR HTO OR tibial surgery

#3 #1 OR #2

#4 tranexamic acid OR AMCHA OR trans‐4‐(Aminomethyl)cyclohexanecarboxylic Acid OR t‐AMCHA OR Cyklokapron OR Amchafibrin OR Exacyl

#5 blood loss

#6 #3 AND #4 AND #5

### *Selection Criteria*

Titles and abstracts were screened for relevant studies and the selection criteria were applied.

Studies that met the PICOS (participants, intervention, comparison, outcome, study design) criteria were included: (i) participants: patients undergoing PAO or HTO; (ii) intervention: tranexamic acid; (iii) comparison: placebo or blank; (iv) outcomes: the primary outcome was blood loss; the secondary outcomes were hemoglobin values, thromboembolic events, transfusion requirements, and wound complications; and (v) study design: randomized control trials (RCT) or cohort studies and case‐control studies.

The exclusion criteria were: (i) low quality studies (<6 stars); (ii) none full‐text; and (iii) non‐lower extremity osteotomy. The study selection was performed by two reviewers (RZ‐Y and CX‐W) independently, and differences between the two reviewers were resolved by their consensus or by the third reviewer (YD‐AM).

### *Data Extraction*

Two reviewers (RZ‐Y and CX‐W) independently extracted data from the involved studies and recorded the items in the data extraction form which was tested before the formal extraction process. The following items were recorded: the author, study year, country, design (RCT, cohort or case–control studies), surgical procedure, route of TXA administration and dose, intervention of the control groups, thromboprophylaxis, demographic characters (sex and age), transfusion cases and units, length of stay, blood loss, hemoglobin values, thromboembolic events, wound complications, and mortality. Differences between the two reviewers were resolved by their consensus or by the third reviewer (YD‐AM). The primary outcome was total blood loss and blood transfusion rate; the secondary outcomes were hemoglobin decline, thromboembolic events, and wound complications.

### *Quality Assessment*

The quality of the included cohort studies were assessed independently by two reviewers according to the Newcastle–Ottawa Quality Assessment Scale (NOS). The full score was 9 stars, and ≥8 stars was high quality, <6 stars was low quality. The assessment items were as follows: representativeness of the exposed cohort, selection of the non‐exposed cohort, ascertainment of exposure, demonstration that outcome of interest was not present at the commencement of study, baseline heterogeneity, assessment of outcome, follow‐up period, and follow‐up rate. The quality of RCT was assessed using the Cochrane Collaboration's tool for assessing risk of bias^21^. Any inconsistencies between the two reviewers (RZ‐Y, CX‐W) were resolved by their consensus or by the third reviewer (YD‐AM).

### *Statistical Analysis*

The analysis process was performed according to the intention‐to‐treat principle. The meta‐analysis was performed with the RevMan 5.3 (The Cochrane Collaboration, Copenhagen, Denmark) and Stata 14.0 software (StataCorp, College Station, Texas， US). The heterogeneity was tested depending on the *P*‐value and *I* ^2^ using the standard χ^2^‐test. *I* ^2^ < 50%, *P ≥* 0.1, was treated there is no statistical evidence of heterogeneity, a fixed‐effects model was adopted; otherwise, a random‐effect model was used. Total blood loss and hemoglobin value changes, as continuous variables, were assessed as a mean difference (MD) with associated standard deviation (SD) and 95% confidence interval (CI). Thromboembolic events, transfusion cases, and wound complications were dichotomous variables, and relative risk (RR) and 95% CI were calculated.

Planned subgroup analyses were performed according to the surgical procedure (PAO *vs* HTO). Funnel plots were used to assess the publication bias and sensitivity analysis was performed to assess the robustness.

## Results

### *Search Results*

**Figure 1:**
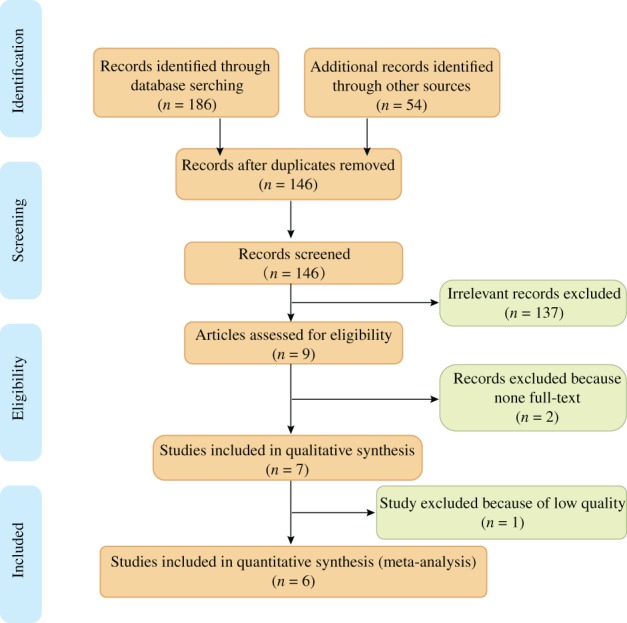
Flow chart of inclusion and exclusion for included studies.

A total of 240 studies were identified after the initial search. After removing the duplicates and screening the abstracts and full texts, finally six cohorts with a total of 665 patients were involved in our analysis 6, 8, 22, 23, 24, 25. All the included studies were published in full text. The reviewers reached an agreement for study selection by discussion. The process of study search and selection are shown in Fig. 1.

### *Study Characteristics*

Three studies were about PAO, and the other three were HTO. The sample size ranged from 30 to 152, and the average age ranged from 26.3 to 58 years. The average follow‐up time was 30 days. One study reported the use of topical TXA^22^; in five studies intravenous TXA was administered^6^, ^8^, ^23^, ^24^, ^25^. PAO were performed using the Bernese surgical approach and HTO with the medial opening wedge. In four studies patients received 2 g TXA during the operation^8^, ^22^, ^23^, ^24^, and in the other two studies a dose of 10 mg/min/Kg was administered^6^, ^25^. In all HTO studies, symptoms were medial compartment degenerative osteoarthritis, Kellgren–Lawrence II, and >5° varus alignment. HTO operations were performed under spinal anesthesia and PAO were under general anesthesia, the mean arterial pressure of both procedures was 60–70 mm Hg. All included studies reported the primary outcomes. The characteristics of the included studies are shown in Table 1.

**Table 1:** The characteristics of the included studies

Study	Year	Country	Design	Full text	Surgical procedure	Route TXA	Total sample	Age (mean ± SD)
Wassilew^6^	2015	Germany	Non‐RCT	Yes	PAO	IV	96	29.6 ± 8.9
Palanisamy^8^	2018	Korea	Non‐RCT	Yes	HTO	IV	152	57.4 ± 5.6
Suh^22^	2017	Korea	Non‐RCT	Yes	HTO	Topical	30	58 ± 5.9
Bryan^23^	2015	America	Non‐RCT	Yes	PAO	IV	137	26.3 ± 8.9
Wingerter^24^	2015	America	Non‐RCT	Yes	PAO	IV	100	27.5 ± 8.3
Kim^25^	2018	Korea	Non‐RCT	Yes	HTO	IV	150	55.3 ± 5.0

HTO, high tibial osteotomy; IV, intravenous; M, mean; non‐RCT, non‐randomized controlled trials; PAO, periacetabular osteotomy; SD, standard deviation; TXA, tranexamic acid.

### *Risk of Bias Assessment*

According to the NOS, two studies scored 8 stars^23^, ^24^, three studies scored 7 stars^6^, ^8^, ^22^, and one study scored 6 stars^24^. The methodological quality assessment according to NOS is presented in Table 2. The “Egger test” was performed to evaluate the publication bias. The results of the funnel plot “Egger test” (*P* = 0.628) indicated a low risk of publication bias. However, publication bias could not be excluded completely, as this meta‐analysis did not include enough studies, so the reliability of these assessments was not very strong.

**Table 2:** Quality assessment according to the Newcastle–Ottawa Quality Assessment Scale (NOS) for cohort

NOS items	Bryan^23^	Suh^22^	Palanisamy^8^	Wassilew^6^	Wingerter^24^	Kim^25^
Representativeness of the exposed cohort	1	1	1	1	1	1
Selection of the non‐exposed cohort	1	1	1	1	1	1
Ascertainment of exposure	1	1	1	1	1	1
Demonstration that outcome of interest was not present at start of study	1	1	1	1	0	1
Comparability of cohorts on basis of design or analysis	2	2	2	2	1	1
Assessment of outcome	0	0	1	0	1	0
Was follow‐up long enough for outcomes to occur	1	1	0	1	1	1
Adequacy of follow up of cohorts	1	0	0	0	0	1
Total score	8	7	7	7	6	7

### *Outcomes of the Meta‐analysis*

#### *Perioperative Total Blood Loss*

**Figure 2:**
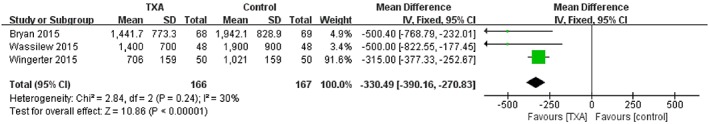
Comparison of perioperative total blood loss of patients undergoing PAO between TXA and control groups. The pooled results showed that TXA could reduce perioperative blood loss in PAO (*P* < 0.001). PAO, periacetabular osteotomy; TXA, tranexamic acid.

**Figure 3:**
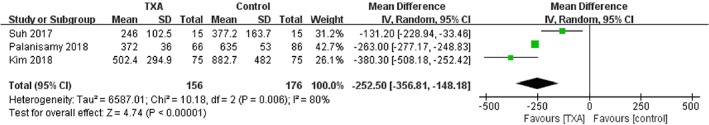
Comparison of perioperative total blood loss of patients undergoing HTO between TXA and control groups. The pooled results showed that TXA could significantly reduce perioperative blood loss in HTO (*P* < 0.001). HTO, high tibial osteotomy; TXA, tranexamic acid.

A total of six studies reported data related to perioperative total blood loss^6^, ^8^, ^22^, ^24^. The random effects model was used for heterogeneity among HTO studies (*I* ^2^ = 80%, *P* = 0.006) and the fixed effects model was used for PAO studies (*I* ^2^ = 30%, *P* = 0.24). The pooled results showed that TXA could significantly reduce perioperative blood loss in both PAO (WMD, −330.49; 95% *CI*, −390.16 to −270.83; *P* < 0.001) (Fig. 2) and HTO (WMD, −252.50; 95% *CI*, −356.81 to −148.18; *P* < 0.001) (Fig. 3).

#### *Hemoglobin Decline*

**Figure 4:**
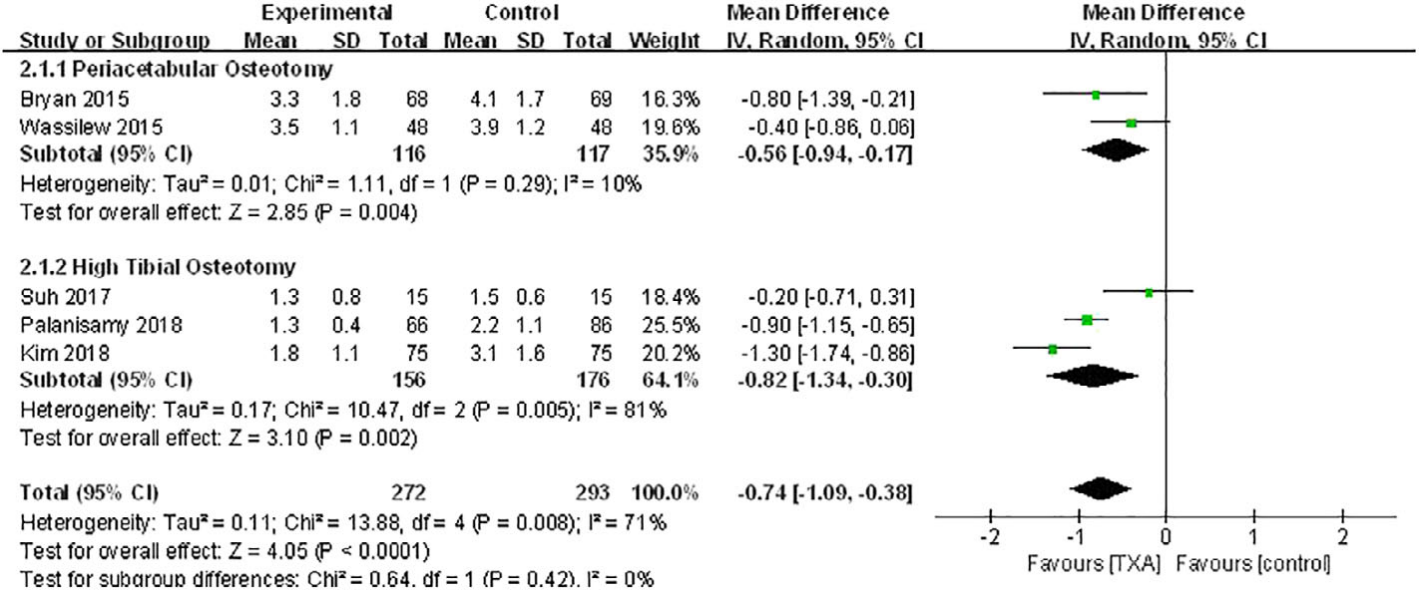
The subgroup analysis according to different surgical procedures of postoperative hemoglobin decline between TXA and control groups. The results indicated that TXA was effective for both periacetabular osteotomy (*P* = 0.004) and high tibial osteotomy (*P* = 0.002). TXA, tranexamic acid.

Changing hemoglobin value was reported in five studies: one study reported the hemoglobin decline for the first day postoperation^23^, two studies reported the hemoglobin decline for the second day postoperation^8^, ^25^, and two studies provided data for the sixth day after operation^6^, ^22^. Heterogeneity existed among the included studies (*P* = 0.008; *I* ^2^ = 71%), which may be attributed to the different surgical procedures; hence the random effects model was used. The combined results indicated that TXA could reduce postoperative hemoglobin loss significantly compared with the control group (WMD, −0.74; 95% *CI*, −1.09 to −0.38; *P <* 0.001) (Fig. 4). The subgroup analysis indicated that TXA was effective for both PAO (WMD, −0.56; 95% *CI*, −0.94 to −0.17; *P* = 0.004) and HTO (WMD, −0.82; 95% *CI*, −1.34 to −0.30; *P* = 0.002).

#### *Blood Transfusion Rate*

**Figure 5:**
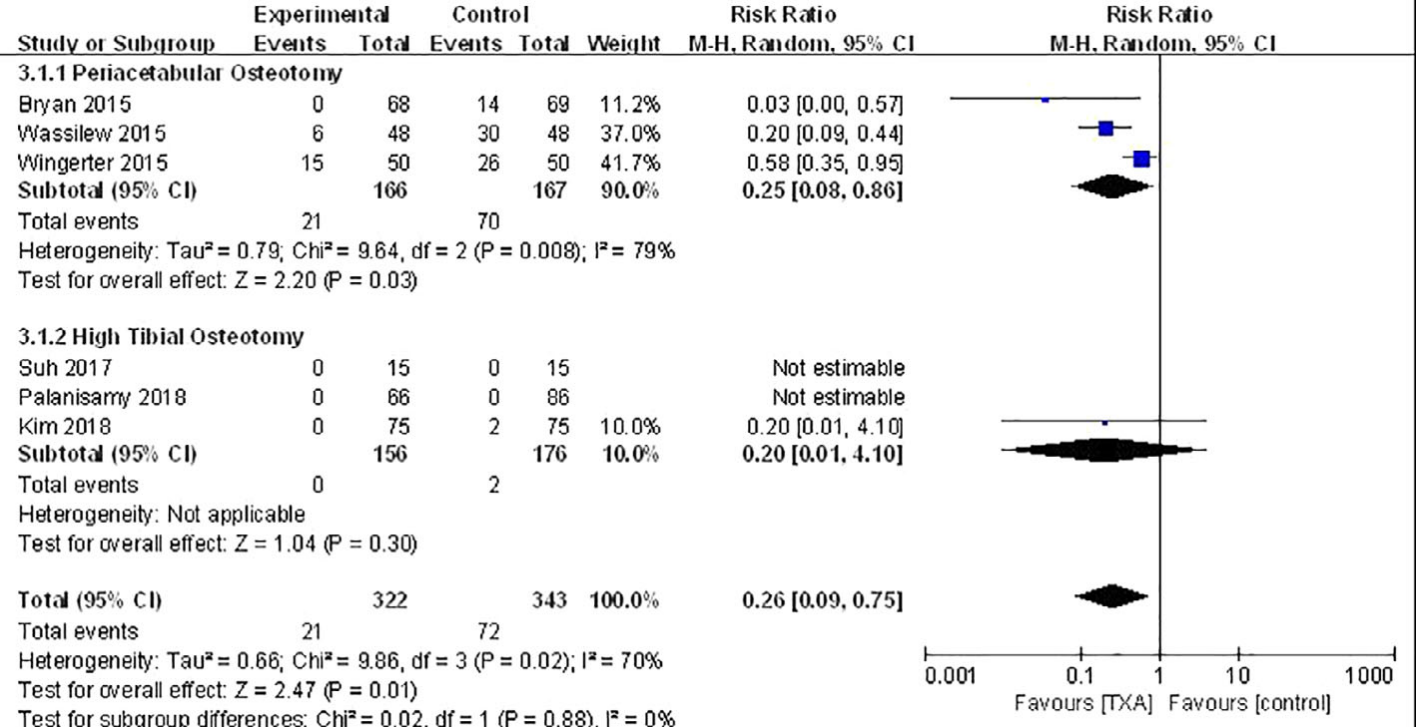
The subgroup analysis according to different surgical procedures of blood transfusion rate between TXA and control groups. The pooled results showed that TXA could reduce the blood transfusion rate for periacetabular osteotomy (*P* = 0.03) but had no effect on high tibial osteotomy (*P* = 0.30). TXA, tranexamic acid.

Blood transfusion rates are shown in all studies and the heterogeneity between studies was significant (*P* = 0.02; *I* ^2^ = 70%); this heterogeneity might be attributed to different surgical procedures. Using the random effects model, we obtained the results that the transfusion rate of PAO was different in TXA compared with the control group (RR, 0.25; 95% *CI*, 0.08 to 0.86; *P* = 0.03), but there was no significant difference for HTO in the TXA group compared with the control group (RR, 0.20; 95% *CI*, 0.01 to 4.10; *P* = 0.30) (Fig. 5).

#### *Wound Complications*

**Figure 6:**
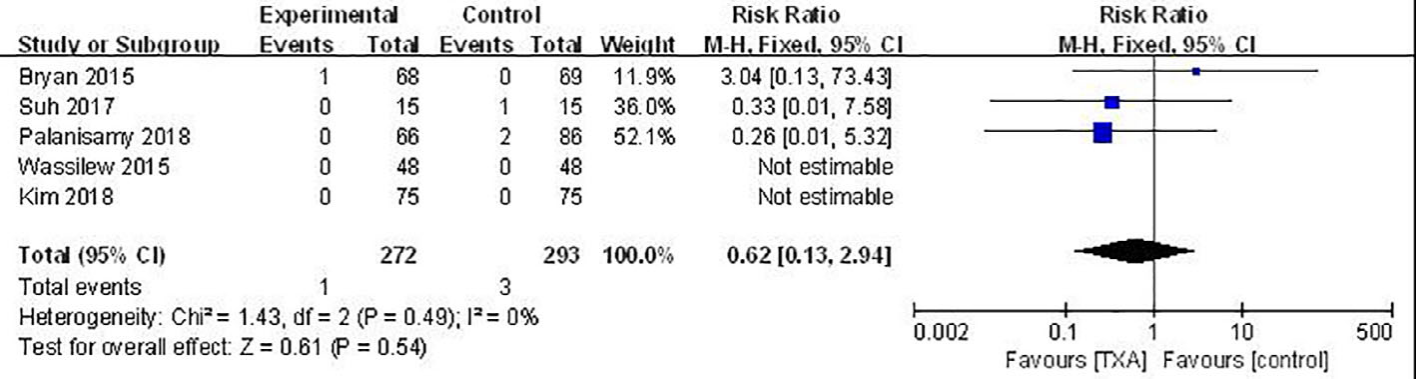
Comparison of wound complications between TXA and control groups. The combined results indicated there was no difference between the two groups (*P* = 0.54). TXA, tranexamic acid.

Five studies reported wound complications; there was low risk of heterogeneity (*P* = 0.49; *I* ^2^ = 0%)^8^, ^22^, ^23^, ^24^, ^25^. The combined results (RR, 0.62; 95% *CI*, 0.13 to 2.94; *P* = 0.54) indicated that there was no significant difference in wound complications in the TXA group compared with the control group (Fig. 6).

#### *Sensitivity*

Each individual study was removed from the model to estimate the robustness of pooled results, and there was no evidence that removal of any single study changed the conclusion that TXA could reduce the perioperative total blood loss and hemoglobin decline.

## Discussion

### *Significance and Major Finding*

As far as we know, this is the first meta‐analysis to evaluate the efficacy and safety of TXA in patients undergoing lower extremity osteotomy. A total of 665 patients from six non‐RCT were involved in this meta‐analysis, and the combined results revealed that TXA could significantly reduce perioperative total blood loss. This is consistent with the evidence that TXA can reduce total blood loss in arthroplasty, spine and trauma surgery^26^, ^27^, ^28^, ^29^, ^30^, ^31^. However, blood loss in PAO is different from that in selective total hip arthroplasty, because of the acetabulum and the pelvis being rich vascular territories and the long duration of the operation. Therefore, the surgeries are associated with heavy total blood loss. Owing to lack of soft tissue coverage, there is greater risk of hematoma and superficial skin infections in HTO. It is important to evaluate the effectiveness of TXA in PAO and HTO, but no systemic review and meta‐analysis has been performed regarding this issue. Thus, there is a requirement for an evidence base to help orthopaedists make clinical decisions. This meta‐analysis indicates that the application of TXA is effective in reducing blood loss and postoperative hemoglobin decline in patients undergoing PAO but has no effect on hemoglobin decline for HTO.

The effect in reducing blood loss of TXA was the most powerful in the first 24 h after surgery. Our finding agrees with Gausden *et al*., who reported that TXA has the strongest hemostatic effect in the first 24 h for trauma surgery as its half‐life period is approximately 3 h^5^. This meta‐analysis found that TXA had no effect on reducing the transfusion rate for HTO. This is different from other research which reported that TXA could reduce the risk of transfusion in selective arthroplasty^26^, ^27^. Two factors may lead to this situation: one is that the quantity of involved studies or patients was not large enough, so the result might have some biases; the other is that the hemostatic effect of TXA was much weaker compared to the heavy bleeding. As the half‐life period of TXA is 3 h, all the included studies had TXA administered before or during the operation; there was no TXA postoperation. However, our finding agrees with Lack *et al*., who reported that TXA had no significant effect in transfusion rate in comparison to placebo in patients undergoing acetabular fracture surgery^32^.

In consideration of increasing the risk of thromboembolic events, many surgeons refuse to give TXA to PAO or HTO patients. All of the included studies reported the thromboembolic events (deep venous thrombosis and pulmonary embolism), and only one study reported that there was a difference between the TXA group (2/66) and the control group (1/68)^23^; other studies reported that there were no thromboembolic events in both TXA and control groups. The pooled result suggested that there was no difference in TXA and control groups. A similar result was seen by Lack *et al*. in a RCT of 88 patients in the USA, that TXA did not increase the risk of venous thromboembolism in acetabular fracture surgery compared with placebo^31^. Wu *et al*. also reported that TXA was safe in total knee arthroplasty^33^. Moreover, each of the included studies failed to detect a significant difference in the rate of thromboembolic events, no matter whether the patients were given anticoagulant therapy or not.

### *Limitations*

Our study has several limitations. First, all the included studies were cohort studies, so there might be more unmeasured and unknown confounders and weaker quality compared with prospective randomized controlled trials. Second, the quantity of studies and patients was not large enough, although we endeavored to search as many databases as possible. To minimize the bias and strengthen the evidence as much as possible, we performed subgroup and sensitivity analyses. Finally, the techniques of the surgeons, the threshold for blood transfusion, and the underlying rate of thromboembolic events may vary widely based on different studies, making these results potentially less applicable to different countries.

### *Conclusions*

Based on the current evidence, this meta‐analysis suggests that TXA reduces total blood loss in both PAO and HTO. In addition, TXA has no relationship with thromboembolic events, so it is safe in PAO and HTO. However, the combined result indicates that TXA does not reduce the transfusion rate or wound complication for HTO, so TXA might be unwarranted for routine use for HTO. Due to the limitations of our study, more well‐designed prospective randomized controlled trials are needed to clarify the effects and safety of TXA in PAO or HTO.
